# Association between frequency of fried food consumption and resilience to depression in Japanese company workers: a cross-sectional study

**DOI:** 10.1186/s12944-016-0331-3

**Published:** 2016-09-15

**Authors:** Eisho Yoshikawa, Daisuke Nishi, Yutaka J. Matsuoka

**Affiliations:** 1 Department of Neuropsychiatry, Nippon Medical School Tama Nagayama Hospital, 1-7-1 Nagayama Tama City, Tokyo, 206-8512 Japan; 2 Department of Neuropsychiatry, Nippon Medical School, 1-1-5 Sendagi, Bunkyo, Tokyo, 113-8602 Japan; 3 Department of Mental Health Policy and Evaluation, National Institute of Mental Health, National Center of Neurology and Psychiatry, 4-1-1 Ogawahigashi-cho, Kodaira, Tokyo, 187-8553 Japan; 4 Department of Public Mental Health Policy, Graduate School of Medicine, The University of Tokyo, Tokyo, 187-8553 Japan; 5 Division of Health Care Research, Center for Public Health Sciences, National Cancer Center, 5-1-1 Tsukiji, Chuo-ku, Tokyo, 104-0045 Japan; 6 Department of Psychiatry, National Disaster Medical Center, 3256 Midoricho, Tachikawa, Tokyo, 190-0014 Japan

**Keywords:** Westernized diet, Fried food consumption, Depressive symptoms, Resilience

## Abstract

**Background:**

Long-chain n-3 and n-6 polyunsaturated fatty acids (LC n-3/n-6 PUFA) play important roles in emotional regulation. We previously reported an association between fish consumption, which is major source of LC n-3 PUFA, and resilience to depression, where resilience is the ability to cope with stress in the face of adversity. Although the traditional Japanese dietary pattern of high fish consumption is associated with low depressive symptoms, the current Japanese diet pattern has become westernized. Westernized diets contain excessive amounts of LC n-6 PUFA due to high intake of vegetable oils commonly used in fried food and are associated with risk of depression. The aim of this study was to examine the association between frequency of fried food consumption and resilience to depression.

**Methods:**

Participants were 715 Japanese company workers. The Center for Epidemiologic Studies Depression Scale (CES-D) was used to measure depressive symptoms, and the 14-item Resilience Scale (RS-14) was used to measure resilience. Frequency of fish and fried food consumption was assessed using a self-report questionnaire based on the Food Frequency Questionnaire. Regression analyses using Preacher and Hayes’ bootstrap script were used to adjust for demographic factors, frequency of physical exercise, and fish consumption.

**Results:**

Significant associations were identified between frequency of fried food consumption and total CES-D score (path c, B = 0.72; *P* < 0.01), between frequency of fried food consumption and total RS-14 score (path a, B = −1.73, *P* < 0.01), and between total RS-14 score and CES-D score (path b, B = −0.35; *P* < 0.01). The association between fried food consumption and total CES-D score was not significant when we controlled for RS-14 score. Bootstrapping results showed that there was a significant positive indirect association between frequency of fried food and CESD score through RS-14 (95 % bias-corrected and accelerated confidence interval = 0.34 to 0.92).

**Conclusion:**

Frequency of fried food consumption was associated with lower resilience to depression. Further nutritional interventional studies to increase resilience and prevent depression are warranted.

## Background

In 1998, Hibbeln reported a negative association between the prevalence of depression and fish consumption in a cross-national analysis [1]. Japan had the highest level of fish consumption and the lowest prevalence of depression [1]. Furthermore, the Japanese diet was associated with low depressive symptoms in both a large cohort study of Japanese workers and a cross-sectional study [2, 3]. The traditional Japanese diet has recently become more westernized. Consumption of fats and oils in 2010 was more than four times that in the 1950s, whereas fish consumption had not changed substantially [4].

Westernized diets have been demonstrated to elevate the risk of depression [5–7], perhaps due to too much linoleic acid, a long-chain n-6 polyunsaturated fatty acid (LC n-6 PUFA) found in vegetable oils and processed foods that are consumed in high quantities in the current westernized diet [8]. An imbalance in the intake of LC n-6 PUFAs and LC n-3 PUFAs may affect emotional regulation and may cause mental disorders, including depression [9]. Fried foods cooked with large amounts of vegetable oil are among the most common westernized foods in Japan.

We previously reported an association between fish consumption and resilience to depression [10]. Resilience, which is generally defined as the ability to cope with stress in the face of adversity [11], is important for preventing depression. Resilience has been associated with regulation of emotions [12–14] and negatively associated with depression [15]. Therefore, frequent fried food intake may affect depressive symptoms by attenuating resilience. The aim of this study was to investigate the association between the frequency of fried food consumption and resilience to depression.

## Methods

### Participants and procedures

This study was conducted by using a database which was collected in a previous study [10, 16]. We approached 2159 workers at six separate worksites of a large company located in an urban area of Japan. Among them, 741 (34.3 %) agreed to participate in the study. Next, full data, with no missing responses to items related to the subscales used in this study, were available for 715 participants. Data from these 715 participants were included in the analysis among them.

### Measures

We collected data on sex, marital status (married or not), educational attainment (university/college graduate or not), and job status (management position or not).

Frequency of fish consumption was measured using the following question: “How often do you usually eat fish or fish meals such as *Sashimi* (raw fish) and/or *Yakizakana* (grilled fish)? Please consider the last six months.” The following question assessed the frequency of fried food consumption: “How often do you usually eat fried food? Please consider the last six months.” Six response options were given for each question: almost never, 1–3 times/month, 1–2 times/week, 3–4 times/week, 5–6 times/week, and every day.

The CES-D is used to assess depressive symptoms [17] and consists of 20 items. The total score ranges from 0 to 60, with a higher score indicating more severe depression. The Japanese version has been shown to be a reliable and valid instrument [18].

Resilience was assessed with the 14-item Resilience Scale (RS-14). Each item is rated on a 7-point Likert scale (total score range, 14–98), with a higher score indicating more resilience [19]. The RS was developed based on a qualitative study of people who had adapted successfully after experiencing a recent loss, such as loss of a spouse, health, or employment [19–23]. The Japanese version has been shown to be valid and reliable [11].

Physical exercise was evaluated by asking, “The next question is about your physical exercise habits. In the last six months, how often did you do relatively hard exercise for more than 20 min, such as jogging or running, cycling, aerobics, or stepping exercise?” The response options were as follows: 1) never, 2) 1–3 times a month, 3) 1–2 times a week, and 4) 3 or more times per week.

### Statistical analysis

All statistical analyses were performed with SPSS, version 23 (SPSS Inc., Chicago, IL). Alpha levels were all set at *P* < 0.05 (two-tailed). To test the mediation model and assess any indirect association, we conducted regression analyses using Preacher and Hayes’ bootstrap script [24] (*n* = 5000 bootstrap samples), which can handle nonparametric data. The bias-corrected and assessed confidence intervals (BCACI) were set at 95 %. The use of 95 % confidence intervals is equivalent to testing for significance at the level of α = 0.05.

**Fig. 1:**
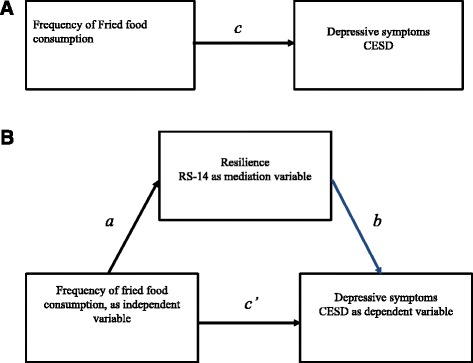
**A** Illustration of a direct association. Path *c* represents total association of frequency of fried food consumption on the total score of the Center for Epidemiologic Studies Depression (CESD). **B** Illustration of a hypothesized model. Path a represents the association of frequency of fried food consumption on the total score of 14-item Resilience Scale (RS-14), the proposed mediator. Path b represents the association of the total score of RS-14 on total score of CESD partialling out the effect of dietary factor. Path c’ is the direct association of dietary factor on total score of CESD partialling out the association of the total score of RS-14. The indirect association of dietary factor on total score of CESD through total score of RS-14 is the product of a and b, which is tested with the bootstrap confidence interval (CI) obtained through the bootstrapping method

Figure 1 a and b illustrate the hypothesized mediation model that fried food intake affected depression (the total CES-D score) by attenuating resilience (the total RS-14 score). The dependent variable was total CES-D score, the independent variable was the frequency of fried food consumption, and the mediator was RS-14 score. Age, sex, educational attainment, marital state, job status, physical exercise, and frequency of fish consumption were used as control variables.

## Results

All 715 participants were Japanese. Mean age ± standard deviation (SD) was 39.9 ± 9.4 years (range = 21–66 years), 596 (83.4 %) were men, and 466 (65.2 %) were married. Most (590, 82.5 %) had graduated from college or university. Regarding working status, 67 (9.4 %) were in management positions. Mean scores ± SD on the five parameters assessed were 63.5 ± 11.0 on RS-14, 10.5 ± 7.5 on CES-D, 3.0 ± 0.9 for the frequency of fish consumption score, 2.0 ± 1.1 for the frequency of physical exercise, and 3.6 ± 1.1 for the frequency of fried food consumption score. The scores of 3.0 and 3.6 here means that, on average, participants ate fish 1–2 times per week and fried food 3–4 times per week.

As shown in Table 1, there were significant associations between frequency of fried food consumption and total CES-D score (path c, B = 0.72; *P* < 0.01), between frequency of fried consumption and total RS-14 score (path a, B = −1.73, *P* < 0.01) and between total RS-14 score and CES-D score (path b, B = −0.35; *P* < 0.01). When controlling for the effects of total RS-14 score, there was no longer a significant association between fried food consumption and total CES-D score. When the size of the indirect association of frequency of fried food on CESD score through RS-14 score was estimated, the bootstrap 95 % CI did not include zero (95 % BCACI = 0.34 to 0.92), which indicated that the indirect association was significant.

**Table 1:** Mediation analysis and bootstrap results for the resilience-mediated relationship between fried food consumption frequency and depression

	B	SE	t	*P*	BCACI
a path^a^	−1.73	0.39	−4.46	<0.01	
b path^a^	−0.35	0.02	−15.82	<0.01	
c' path^a^	0.11	0.23	0.485	0.63	
c path^a^	0.72	0.27	2.71	<0.01	
ab (c-c') path					[0.34 0.92]

*SE* Standard error, *BCACI* bias-corrected and assessed confidence interval

^a^Regression coefficient between each pair of variables corresponding to the ends of the path indicated with the symbol in Fig. 1

## Discussion

The findings of this study are consistent with the hypothesis that more frequent fried food consumption decreases resilience, and is therefore associated with developing depression. Decreasing the intake of fried food, which contains high levels of linoleic acid from vegetable oils, and consuming fish, which is major source of LC n-3 PUFA, may be important for promoting resilience to depression.

However, this was a cross-sectional study, so we could not determine causal relationships between the factors. Resilience has been reported to be associated with health-promoting behavior or compliance with treatment of physical illness [25, 26]. Another possible association is that low resilience promotes fried food consumption and results in depressive symptoms. However, in this case, an alternative mediation analysis, which was conducted to enter resilience as independent variable, fried food consumption as a mediator variable, and depressive symptoms as dependent variables, did not demonstrate significant indirect association (data not shown). Diet is a possible indicator of overall lifestyle patterns and health-related behaviors mutually associated with depressive illness. For example, fried food intake might be associated with a lifestyle factor, such as consuming snack foods and beverages, that has been associated with depression [27]. The possibility exists that residual confounding by such uncontrolled or unmeasured factors may have distorted genuine associations.

Other limitations were as follows. First, most of the participants were men, were highly educated, and worked for a large Japanese company that provides good job security and a relatively good balance of effort and reward. Second, the response rate was not high and therefore the finding may not be widely generalizable. Third, information on frequency of fried food consumption was self-reported, and non-differential misclassification may be inevitable and could attenuate the observed associations. In addition, there are many sources of LC n-6 PUFA other than fried food. A specific food frequency questionnaire for PUFA intake was not used in this study [28].

## Conclusion

The results of the current study suggest that frequency of fried food consumption is associated with lower resilience to depression, independent of frequency of fish consumption. Double-blind, randomized, placebo-controlled nutritional intervention trials on resilience to depression are needed.

## Abbreviations

BCACI: Bias-corrected and accelerated confidence interval
CES-D: The center for epidemiologic studies depression
CI: Confidence interval
RS-14: 14-item resilience scale
